# Diagnostic value of a 3-day course of prednisolone in patients with possible rheumatoid arthritis – the TryCort study

**DOI:** 10.1186/s13075-017-1279-z

**Published:** 2017-04-07

**Authors:** Uta Kiltz, Christine von Zabern, Xenofon Baraliakos, Frank Heldmann, Bernd Mintrop, Michael Sarholz, Dietmar Krause, Friedrich Dybowski, Ludwig Kalthoff, Jürgen Braun

**Affiliations:** 1 Rheumazentrum Ruhrgebiet, Claudiusstraße 45, 44649 Herne, Germany; 2 Zeisigwaldklinik Bethanien Chemnitz, Zeisigwaldstraße 101, 09130 Chemnitz, Germany; 3 Rheumatology private office in Gladbeck, Friedrich-Ebert-Straße 2, 45964 Gladbeck, Germany; 4 Rheumatology private office in Herne, Claudiusstraße 45, 44649 Herne, Germany; 5 Rheumatology private office in Bochum, Gudrunstrasse 56, 44791 Bochum, Germany

## Abstract

**Background:**

In patients with tender and swollen finger joints, the differential diagnosis between rheumatoid arthritis (RA) and osteoarthritis (OA) of the hands can be initially difficult. This prospective study (the TryCort study) was performed to study the diagnostic value of prednisolone in differentiating between RA and hand OA. We present the results of this potentially diagnostic test in patients with possible RA in daily clinical practice by demonstrating the results of a pilot and a validation part of this ‘prednisolone test’ (pred-test).

**Methods:**

We investigated the response to a 3-day course of 20 mg of prednisolone in patients with suspicion of RA. All patients received 1 g of paracetamol per day for 5 days for pain relief. On days 3–5, a morning dose of 20 mg of prednisolone was added. Hand pain was quantified on a 0–10 Numerical Rating Scale, and the subjective percentage of improvement (0–100%) was recorded. Thresholds for response to prednisolone were investigated in a pilot phase with differentiation in response between patients with RA and patients with OA of the hands, both with pain in the hands ≥4. In a validation phase, the best differentiating cut-off of the pilot phase was applied to discriminate responders from non-responders in consecutive patients with hand pain ≥4 referred because of suspected RA. Final diagnoses were made by the expert upon re-examination at week 12. Primary outcomes were the sensitivity and specificity of a positive test in relation to the diagnosis.

**Results:**

A percentage of 40% subjective improvement of pain in the hands on day 3 discriminated best between RA and OA in the pilot phase. Among 95 patients with complete data in the validation phase, RA was diagnosed in about 50%. Patients with RA had more swollen joints, higher C-reactive protein levels and slightly higher Health Assessment Questionnaire scores. The pred-test was positive in 42.1% of all patients (40 of 95). The median percentage of improvement on day 5 was higher in RA than in non-RA: 50% (IQR 30–60%) vs. 20% (IQR 10–30%) (*p* < 0.001). The sensitivity and specificity of the pred-test were 0.6 (95% CI 0.5–0.8) and 0.8 (95% CI 0.7–0.9), respectively, and the positive and negative predictive values were 0.77 and 0.70, respectively.

**Conclusions:**

To our knowledge, this is the first evaluation of the widely used pred-test that has ever been performed. The pred-test had a moderate sensitivity and a good specificity. We conclude that rheumatologists may use this test in unclear clinical situations to better differentiate between inflammatory and other conditions.

**Trial registration:**

ClinicalTrials.gov identifier: NCT01395251. Registered on 14 Jul 2011.

EudraCT number: 2011-002633-19. Registered on 21 Dec 2011.

## Background

Joint pain of the hands is among the most frequent symptoms and reasons for admission in rheumatology. The biggest problem is arthralgia of the hands caused by rheumatoid arthritis (RA) and/or hand osteoarthritis (OA) [1]. In patients with tender and swollen finger joints, the differential diagnosis between RA and OA can be difficult, especially in relatively early disease stages and also when the diseases overlap, and there is no evidence that RA protects against OA or vice versa. Current classification criteria are of limited help because of their poor diagnostic utility in early and more advanced disease stages [2, 3]. Similarly, the specificity of the RA classification criteria was 0.61, suggesting that 39% of the patients have a diagnosis other than RA [4]. In addition, many patients are initially diagnosed with undifferentiated arthritis, and some of these patients will fulfil RA classification criteria at later time points [5].

For the differential diagnosis between RA and OA, the main question is usually whether the cause of arthralgia is related to inflammation or to degenerative mechanisms. However, even this differentiation is not so straightforward anymore, because magnetic resonance imaging (MRI) findings of synovitis and osteitis have also been described in patients with OA [6]. Furthermore, involvement of the distal interphalangeal joints may indicate OA rather than RA. However, even this important clinical feature may be misleading because the patient may well have both diseases [7]. Clinically, there are different ways to potentially answer the question of RA vs. OA – a clinically relevant question because of the largely different therapeutic options [6, 8]. Of potential help for the differential diagnosis are patient history and the presence of morning stiffness for >30 minutes, the joint pattern in the physical examination and/or the patient history, and the obvious presence of swelling and tenderness of joints. When taking technical procedures into account, the measurement of biomarkers of inflammation, erythrocyte sedimentation rate (ESR) and C-reactive protein (CRP) may be useful to differentiate OA from RA. For RA, there are even specific tests available, such as anti-citrullinated peptide antibody (ACPA), which are also believed to be of pathophysiological importance in terms of their pathogenetic mechanism, which in part is also connected to smoking habits of patients [8]. Finally, imaging procedures may be helpful, especially those with the capacity to visualize inflammation. MRI and ultrasound techniques are now increasingly used to search for arthritis and synovitis in both RA and OA [9, 10]. However, clinical diagnosis is still considered the gold standard for a diagnosis of these two most frequent rheumatic diseases.

However, there are patients and clinical situations where even the totality of the evidence these tests and examinations provide cannot definitively answer the clinical question posed. At this point in time, many rheumatologists are accustomed to just performing a short therapeutic attempt with glucocorticoids (GCs). The effect of GCs in reducing the inflammatory burden of arthritic conditions has been well known for almost 70 years [11]. GCs still play an important role in international recommendations for the treatment of RA [12, 13], and they are still frequently used in daily practice [14]. The mechanisms of action are now far better understood [15, 16]. The basic idea is that RA is due to inflammation, whereas OA is either not based on the same pathophysiology, or at least not to the same degree. However, a short therapeutic attempt with GCs is a clinical test whose diagnostic value has never been evaluated. This prospective study (the TryCort study) was performed to evaluate the diagnostic value of prednisolone in differentiating between RA and hand OA.

## Methods

### Study design

The TryCort study was performed as a monocentric, prospective clinical study to add another tool to improve the diagnosis of RA. The study was planned in two phases: a pilot and a validation study. The pilot part was designed as a proof-of-concept study to investigate the effect of a 3-day treatment intervention with 20 mg of prednisolone (the ‘pred-test’) in patients with established RA and hand OA. In detail, we wanted to determine the ‘cut-off’ for response, and we also aimed to gain first insight into the percentage of responders. The validation study was designed to test the diagnostic utility of the pred-test and to confirm the threshold of the treatment effect.

The ethics committee of the University of Muenster approved the study protocol, and all patients gave informed consent before participation. This study is registered with EudraCT (2011-002633-19).

### Study populations

Consecutive patients aged >18 years and <80 years presenting to our hospital or two private practice offices of cooperating rheumatologists were included in the study between January 2012 and November 2014. In the pilot phase, patients with pain in their fingers and/or hands and either an established diagnosis of RA or an established diagnosis of hand OA according to the treating rheumatologist were eligible for enrolment into this first phase. Patients with RA had to fulfil the American College of Rheumatology (ACR)/European League Against Rheumatism (EULAR) 2010 criteria for RA [2], and patients with OA had to fulfil the ACR classification criteria for OA [3].

In the validation phase, patients with suspicion of RA were eligible for enrolment into this phase. Suspicion of RA was defined as chronic pain in fingers and hands lasting >6 weeks without other causality. The presence of pain in finger joints was mandatory; pain in the hands (palm and wrist) was not. Patients with hand OA fulfilling ACR classification criteria for OA but who were positive for ACPA were not included in the trial. We excluded patients with triggering factors for inflamed joints, such as tophi, psoriasis or accidents.

For both groups, the degree of pain had to be ≥4 on a 0–10 Numerical Rating Scale (NRS). Patients receiving concomitant treatment with oral GCs were not eligible. In addition, patients who had been treated with oral GCs of any dose within the 2 weeks before their baseline visit were not included in the trial. In addition, patients with uncontrolled diabetes mellitus, hypertension and glaucoma, as well as those with pregnancy or desire to become pregnant, were not eligible.

### Interventions

**Fig. 1:**
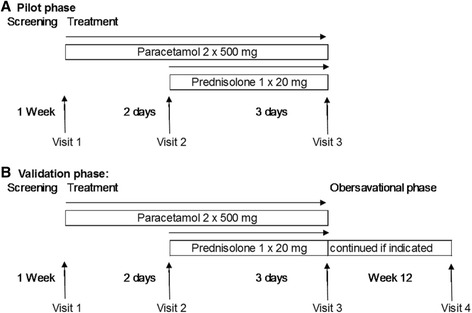
Study design of the pilot (**a**) and validation (**b**) phases

All patients received 1 g of paracetamol/day for 5 days (days 1–5) during the pilot and validation study to provide a background of pain reduction for ethical reasons and comparability (Fig. 1). Rationale for paracetamol was the low analgesic potency and the absence of an anti-inflammatory effect so that medication could serve as background medication without disturbing response to GC therapy. Patients with an insufficient reduction of pain could ask for an additional dose of paracetamol up to a maximum of 2 g of paracetamol/day. Other analgesics or non-steroidal anti-inflammatory drugs were not allowed and were discontinued before the start of the study.

On days 3–5, a morning dose of 20 mg of prednisolone was added. An intervention phase lasting about 3 days was considered sufficient because prednisolone mediates a quick anti-inflammatory response, but prednisolone requires a 3-day interval to influence inflammation in joints. Patients in the validation phase received the same trial medication (paracetamol on days 1–5, prednisolone on days 3–5) as patients in the pilot phase. Intervention on days 1–5 in the validation phase was followed by an observational phase up to week 12. In this observational phase, the patients did not receive study medication but might receive therapy with disease-modifying anti-rheumatic drugs (DMARDs) if RA had been diagnosed. The choice of DMARD therapy was not pre-defined in the study protocol, and the decision was up to the treating rheumatologist (Fig. 1). Patients underwent re-examination by a rheumatologist in week 12 to confirm the diagnosis made on day 5. Re-examination consists of history, physical examination, assessment of patient-reported outcomes and safety signals and CRP measurement.

### Outcome assessments

Pain in the hands was assessed on the basis of an NRS score of 0–10 (0 = no pain, 10 = severe pain). The improvement of pain was determined on a percentage scale of 0–100% (0 = no improvement, 100% = optimal improvement). The 68-joint count for swelling and tenderness was performed by one rheumatologist blinded to laboratory and imaging data to exclude an influence of these results on the physical examination.

Disease activity was measured using the 28-joint Disease Activity Score (DAS28) [17] and the Rheumatoid Arthritis Disease Activity Index (RADAI) [18]. Functional disability was assessed by the Funktionsfragebogen Hannover (FFbH) score, which strongly correlates with the Health Assessment Questionnaire (HAQ) [19]. Values of FFbH were converted into HAQ values by the published formula: HAQ score = 3.16 − (0.028 × FFbH score). Grip strength was evaluated by using a standard dynamometer. All clinical measurements were recorded at each visit (days 1, 3 and 5 and week 12) in the afternoon. Time of day was important to assess treatment response of the morning prednisolone dose. Laboratory parameters measured included CRP measured at days 1 and 5 and week 12, and ACPA and rheumatoid factor measured at day 1.

Furthermore, all patients underwent the usual diagnostic procedures performed in our centre, independent of the study design. The evaluation of conventional radiographs and MRI was performed by an experienced radiologist in our centre. Safety assessments were based on reports of adverse events (AEs), routine physical examinations and vital signs, and laboratory test results were documented in each visit. The clinical diagnosis of an independent and experienced rheumatologist after 3 months was used as a gold standard.

### Evaluation

The primary endpoint of the pilot phase was the proportions of patients with RA and patients with OA with positive pred-tests. The pre-defined cut-off of 40% improvement was applied to differentiate between responders and non-responders. Thus, the pred-test was defined as being positive if patients had an improvement of ≥40% on day 5. The primary endpoint of the validation phase was the sensitivity, specificity and predictive value of the pred-test.

### Statistical analysis

Descriptive data are presented as absolute values and their proportions when referring to qualitative variables. Continuous variables are expressed as the mean ± SD or as the median with IQR (25–75% range) where appropriate. The Mann-Whitney *U* test was used to compare the data between subgroups at single time points. A *p* value <0.05 was considered statistically significant. The diagnostic utility of the pred-test was determined by calculating the sensitivity, specificity and positive and negative predicative values. AUC was analysed and graphically plotted by using ROC curve analysis to show performance of the cut-off values. For the statistical analysis, IBM SPSS Statistics version 22 software (IBM, Armonk, NY, USA) was used.

## Results

**Fig. 2:**
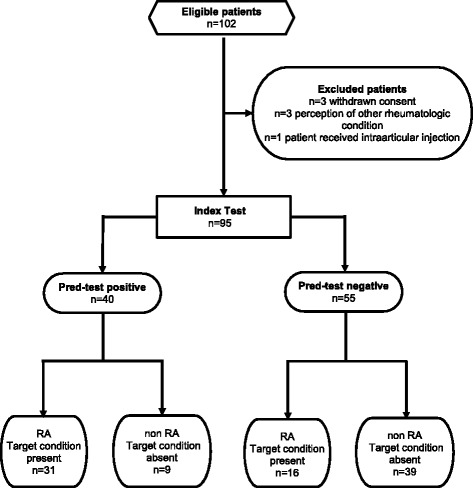
Flow diagram of the validation phase. *RA* Rheumatoid arthritis

A total of 132 patients were eligible for the study, 125 of whom were included in the study: 30 in the pilot and 95 in the validation study (Fig. 2).

### Pilot phase

A total of 15 patients with a confirmed diagnosis of RA and 15 patients with a confirmed diagnosis of OA, respectively, were included. In the RA group, there were 13 women (86.7%), and in the OA group, there were 11 women (73.3%). Demographic and clinical characteristics of the patients are presented in Table 1.

**Table 1:** Demographic characteristics of the patients with rheumatoid arthritis, osteoarthritis in the pilot study and of patients with rheumatoid arthritis and patients without rheumatoid arthritis in the validation study

	Pilot phase		Validation phase	
	RA (*n* = 15)	OA (*n* = 15)	RA (*n* = 47)	Non-RA (*n* = 48)
Mean age, years	59.3 ± 6.3	66.8 ± 10.3	57.6 ± 12.2	54.5 ± 9.2
Female sex	86.7%	73.3%	70.2%	83.3%
Symptom duration, years	4.0 ± 3.5	10.8 ± 13.9	5.3 ± 10.2	4.9 ± 5.7

*OA* Osteoarthritis, *RA* Rheumatoid arthritis

Values are given as mean ± SD

**Fig. 3:**
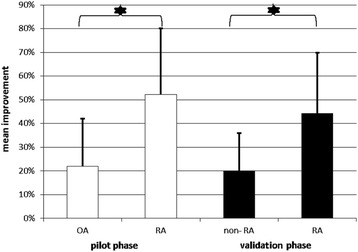
Difference between RA and non-RA regarding the mean improvement of pain in the hands after prednisolone test in the pilot and validation phases. *RA* Rheumatoid arthritis, *OA* Osteoarthritis

The mean age and symptom duration of the patients with OA were somewhat higher than those of patients with RA. The mean pain scores were similar in patients with RA (5.4 ± 1.6) and patients with OA (5.3 ± 1.4). However, patients with RA experienced a greater reduction in pain scores between days 1 and 5 to 2.9 ± 1.8 (RA) and 4.1 ± 1.5 (OA), respectively. The mean percentages of improvement at day 5 (after 3 days of 20-mg prednisolone therapy) were 52.3 ± 27.9% in the RA group and 22.0 ± 20.1% in the OA group (Fig. 3).

The sensitivity and specificity for the cut-offs of subjective improvement of 20%, 30%, 40% and 50% are given in Table 2. The threshold of 40% of subjective improvement was selected as the best-performing cut-off, so the pred-test was defined as being positive if the patient reported a subjective improvement in hand pain ≥40%. Applying this threshold, the pred-test was positive in 11 patients with RA (73.3%) and 4 patients with OA (26.7%) (Table 2) (*p* = 0.012). The sensitivity and specificity for a diagnosis of RA were both 73.3%.

**Table 2:** Diagnostic utility of the different cut-offs

Cut-off of subjective improvement	Pilot phase		Validation phase	
	Sensitivity^a^	Specificity	Sensitivity	Specificity
20%	93.3%	37.5%	79.2%	41.7%
30%	80.0%	66.7%	76.6%	58.3%
40%	73.3%	73.3%	66.0%	81.3%
50%	60.0%	80.0%	57.5%	91.7%

^a^Based on fourfold table

The safety analysis showed that ten patients (30%) had adverse events (AEs), five of them with gastrointestinal complaints which were considered drug-related AEs. No serious AEs were recognized

### Validation phase

A total of 95 patients with pain in their fingers and hands without a clear diagnosis were enrolled in the validation phase and completed the 5 days of the intervention. Of these 95 patients, 78 (82%) participated in the final visit at week 12. According to the judgement of the rheumatologist at week 12, 47 patients were diagnosed with RA and 48 with non-RA. The mean age and symptom duration of the patients were rather similar in patients with RA and subjects with non-RA (Table 1). Patients with RA had more swollen joints, higher CRP levels and comparable HAQ scores assessed on day 1 (Table 3).

**Table 3:** Clinical characteristics of patients with rheumatoid arthritis and non-rheumatoid arthritis in the validation phase

	Day 1			Day 5		
Characteristic	RA (*n* = 47)	Non-RA (*n* = 48)	*p* Value	RA (*n* = 47)	Non-RA (*n* = 48)	*p* Value
Pain	6.1 ± 1.6	5.8 ± 1.6	0.3	3.4 ± 2.3	4.3 ± 1.8	<0.05
Swollen joint count	2.3 ± 2.4	1.4 ± 2.3	0.02	2.3 ± 4.3	1.1 ± 2.0	0.06
Tender joint count	13.0 ± 8.5	13.9 ± 10.5	0.7	12.0 ± 13.1	11.1 ± 8.9	0.8
CRP, mg/dl	0.5 ± 0.6	0.2 ± 0.3	0.03	0.4 ± 0.6	0.2 ± 0.1	0.1
DAS28	3.7 ± 0.8	3.3 ± 0.8	0.1	3.2 ± 1.0	3.1 ± 0.8	0.7
RADAI	5.0 ± 1.4	4.6 ± 1.2	0.2	3.7 ± 1.53	4.0 ± 1.33	0.4
HAQ	1.2 ± 0.6	1.1 ± 0.4	0.8	1.0 ± 0.5	1.1 ± 0.4	0.2
Grip strength, Pascal	40.2 ± 21.8	35.8 ± 17.6	0.4	38.4 ± 22.1	43.4 ± 26.2	0.5

*Abbreviations: CRP* C-reactive protein, *DAS28* 28-Joint Disease Activity Score, *HAQ* Health Assessment Questionnaire, *RA* Rheumatoid arthritis, *RADAI* Rheumatoid Arthritis Disease Activity Index

Values are given as mean ± SD

Assessment: CRP <0.5 mg/dl, DAS28 range 0–10, grip strength range 0–1.0, HAQ range 0–3, pain NRS range 0–10, RADAI range 0–10, swollen joint count range 0–66, Tender joint count range 0–68

Patients with RA experienced a more severe reduction of pain during the treatment course with prednisolone. Similarly, the median percentage of improvement at day 5 was higher in patients with RA than in those with non-RA: 50% (IQR 30–60%) vs. 20% (IQR 10–30%) (*p* = 0.001) (Fig. 3).

**Fig. 4:**
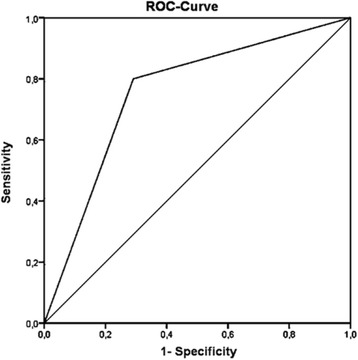
ROC curve of the diagnostic utility of the prednisolone test in the validation phase

Overall, 40 (42.1%) of 95 patients had an improvement of ≥40% on day 5, fulfilling the criteria of a positive pred-test. More patients with RA than patients with non-RA had a positive pred-test (31 patients with RA [77.5%] vs. 9 patients with non-RA [22.5%]; *p* <0.001). The sensitivity of the pred-test for a diagnosis of RA was 0.6 (95% CI 0.5–0.8), and the specificity was 0.8 (95% CI 0.7–0.9). The positive and negative predictive values were 0.77 and 0.70, respectively. The ROC analysis showed an AUC of 0.77 (95% CI 0.65–0.85) (Fig. 4).

The safety analysis showed that 23 patients (24.2%) had AEs, and 16 AEs were considered drug-related (5 headaches, 5 flushings, 2 tachycardia, 2 hypertension, and 2 dizziness). There were no serious AEs.

## Discussion

The TryCort study is the first evaluation of a widely used test, the pred-test, that has ever been performed to systematically investigate its diagnostic value in patients with possible RA. The data document a statistically significant association between positive responses to GCs and a diagnosis of RA. Our hypothesis that more patients with RA than patients with non-inflammatory rheumatic conditions will improve when treated with GCs was clearly confirmed and substantiated in both study phases.

The anti-inflammatory properties of GCs are certainly well established, and they are widely used to treat inflammatory rheumatic conditions such as RA [14, 20]. Accordingly, in a recent early arthritis cohort study, the response to a GC bridging therapy at 2 weeks was clearly predictive of having active disease at 3 months [21]. In contrast to our study, the duration of the intervention was much longer. Furthermore, the main question of our study was different, because we studied the diagnostic utility of GCs to diagnose inflammatory arthritis and to differentiate RA from non-RA.

For many years, rheumatologists have been using a short therapeutic attempt with prednisolone in unclear clinical situations to differentiate between inflammatory arthritis and non-inflammatory conditions. Our results confirm that this test may indeed be useful in this regard. The assumption that systemic GCs do not really work in OA has recently been backed by the results of a study in OA in which patients with OA reported no significant pain reduction after treatment with 5 mg of prednisolone for 4 weeks, although some synovitis had been initially detected by MRI [6]. This is in contrast to RA, where even low doses of prednisolone were shown to be efficacious by reducing disease activity and to even inhibit radiographic progression [14, 22, 23]. Therefore, GCs have even been considered as DMARDs in RA [23], and recent international recommendations have addressed all issues in connection with the use of prednisolone in RA [12, 24, 25]. Of interest, to provide help for the clinical diagnosis, the diagnostic utility of prednisolone has also been studied in patients with chronic obstructive pulmonary disease (COPD) [26], but no meaningful diagnostic value was found. This may be explained by the pathophysiologic differences between COPD and RA.

Could the patients with early arthritis and arthralgia involving hands and feet in our study have had rheumatologic diagnoses other than RA? Several other inflammatory rheumatic diseases, such as psoriatic arthritis, connective tissue diseases and even vasculitides, are known to possibly present with a similar picture [27]. Establishing a differential diagnosis was therefore one of the key elements before including patients in the study. However, because the gold standard of our test was a clinical diagnosis of an independent and experienced rheumatologist after 3 months, we cannot be perfectly sure that we included patients with other inflammatory arthritic conditions of the hand. As stated in the Methods section above, we tried to minimize this bias by performing a standard diagnostic work-up for every patient. Furthermore, many patients have to be classified as having undifferentiated arthritis because they do not fit into one of the well-defined categories. However, patients may well progress from an initial classification of undifferentiated arthritis to RA [5]. In any case, the response to therapy was similar in a large recent study [28].

Because the early classification of RA may be difficult, in this study we decided to rely on an expert’s diagnosis after an observational period of 12 weeks. However, we are aware that even this period of time may not be long enough to finally ascertain a diagnosis of RA. Thus, we cannot exclude that some patients with a diagnosis of possible RA would have been diagnosed differently at later time points. Other diagnoses would include, for example, psoriatic arthritis and undifferentiated arthritis. Because there is some evidence that MRI does not differentiate patients with established RA from other forms of early arthritis, we could not use such results for this purpose [29]. We are aware that symptom duration of approximately 5 years in our patient population (validation cohort) might be a matter of debate. However, in patients with arthralgia but without clear synovitis, diagnosis is often delayed and is even seen in registry cohorts [30].

The ACR/EULAR classification criteria for RA require at least one swollen joint [2]. The data of our study showed that one-third of the patients diagnosed with RA initially had clinically no clear swollen joints (the main inclusion criterion for this study was finger and hand pain) but MRI changes suggestive of synovitis. Because it is now well established that early diagnosis and treatment are critical for patients with RA [8, 31], we think that our study gives a first hint that the response to GCs could become an important component of diagnosing and treating patients with RA at early time points and when the ACR/EULAR criteria are not reliably fulfilled. Of course, the prognostic significance of this test needs to be assessed in studies with a different design.

Our pilot study revealed that a 40% improvement was the best choice between sensitivity and specificity regarding a diagnosis of RA. The ACR 20% improvement criteria (ACR20) are widely used in RA trials [32, 33]. However, their performance related to discrimination is known not to be optimal [34]. Indeed, the ACR50 criteria seem to be superior [35]. Our evaluation for this diagnostic test showed a moderate sensitivity of 65.9% with a good specificity of 81.2%. Thus, our pred-test performed well, but it did not perform perfectly well. Possible reasons are our inhomogeneous patient cohort, lack of an objective gold standard for diagnosis and arbitrarily chosen time point for diagnostic testing. Some of these aspects (e.g., symptom duration) might have had an impact on test accuracy, but this cannot be quantified within our study design.

We are aware that the pred-test without confirmation of other surrogate markers is not helpful in clinical decision-making processes. We therefore recommend use of the test in light of other confirming factors, such as history, physical examination, imaging and laboratory results. Whether the test is useful in the hands of primary care doctors cannot be answered by our study. However, we think that the pred-test can triage patients from primary care to rheumatology specialists.

On one hand, a positive test result will help to identify patients with inflammatory arthritis, especially RA, who are then subject to proper treatment. In case of a negative test result, the likelihood of RA is low. However, a re-evaluation may still be necessary if suggestive symptoms appear. Nevertheless, may a negative test result prevent patients from receiving unnecessary treatment with GCs?

On the other hand, our study also shows that 4 of 15 patients in the pilot phase and 15 of 47 patients diagnosed with RA in the validation phase had a negative test result. One possible explanation for this is that some patients may need higher doses of GCs. Indeed, the Combination Therapy for Rheumatoid Arthritis (COBRA) study showed that a high GC dose of 60 mg/day worked well in many patients with RA [36]. Ever since then, also starting doses of 10 mg/day have been used to reach remission [37, 38], but for maintenance therapy, even dosages <5 mg/day have been successful in some patients [39]. In an ongoing study, the performance of two GC doses were compared in patients with early RA [40]. Different tapering strategies have been proposed [24]. Especially, the questions of which patients may need higher dosages of prednisolone and why deserve further study. GC receptors may have a role in this [16]. Another possibility is that, in addition to RA, there may be other reasons for pain, such as OA, and the patient is unable to differentiate between the two.

## Conclusions

This study shows that the pred-test can support clinical decision-making as a diagnostic aid in differentiating between RA and non-RA. These data also confirm a cut-off point of ≥40% improvement with good sensitivity and specificity. The study also clearly shows that a positive response to prednisolone cannot be taken as evidence that a diagnosis of RA is a given. Further studies are needed to confirm the diagnostic utility of the pred-test with larger patient groups in rheumatology as well as in primary care. Future research can be focused on test accuracy in the light of possible influencing factors.

## Abbreviations

ACPA: Anti-citrullinated peptide antibody
ACR: American College of Rheumatology
ACR20: American College of Rheumatology 20% improvement criteria
ACR50: American College of Rheumatology 50% improvement criteria
AE: Adverse event
COPD: Chronic obstructive pulmonary disease
CRP: C-reactive protein
DAS28: 28-joint Disease Activity Score
DMARD: Disease-modifying anti-rheumatic drug
ESR: Erythrocyte sedimentation rate
EULAR: European League Against Rheumatism
FFbH: Funktionsfragebogen Hannover
GC: Glucocorticoid
HAQ: Health Assessment Questionnaire
MRI: Magnetic resonance imaging
NRS: Numerical Rating Scale
OA: Osteoarthritis
pred-test: Prednisolone test
RA: Rheumatoid arthritis
RADAI: Rheumatoid Arthritis Disease Activity Index
RF: Rheumatoid factor
